# The *Trypanosoma cruzi* pleiotropic protein P21 orchestrates the intracellular retention and *in-vivo* parasitism control of virulent Y strain parasites

**DOI:** 10.3389/fcimb.2024.1412345

**Published:** 2024-06-26

**Authors:** Anna Clara Azevedo Silveira, Nelsa Paula Inácio Uombe, Teresiama Velikkakam, Bruna Cristina Borges, Thaise Lara Teixeira, Vitelhe Ferreira de Almeida, Jhoan David Aguillon Torres, Cecília Luiza Pereira, Guilherme de Souza, Samuel Cota Teixeira, João Paulo Silva Servato, Marcelo José Barbosa Silva, Tiago Wilson Patriarca Mineo, Rosineide Marques Ribas, Renato Arruda Mortara, José Franco da Silveira, Claudio Vieira da Silva

**Affiliations:** ^1^ Instituto de Ciências Biomédicas, Universidade Federal de Uberlândia, Uberlândia, Minas Gerais, Brazil; ^2^ Departamento de Microbiologia, Imunologia e Parasitologia, Universidade Federal de São Paulo, São Paulo, Brazil; ^3^ Faculdade de Odontologia, Universidade de Uberaba, Uberaba, Minas Gerais, Brazil

**Keywords:** *Trypanosoma cruzi*, CRISPR/Cas9, parasite-host interaction, cell invasion, intracellular multiplication, virulence

## Abstract

P21 is a protein secreted by all forms of *Trypanosoma cruzi* (*T. cruzi*) with recognized biological activities determined in studies using the recombinant form of the protein. In our recent study, we found that the ablation of P21 gene decreased Y strain axenic epimastigotes multiplication and increased intracellular replication of amastigotes in HeLa cells infected with metacyclic trypomastigotes. In the present study, we investigated the effect of P21 *in vitro* using C2C12 cell lines infected with tissue culture-derived trypomastigotes (TCT) of wild-type and P21 knockout (TcP21^−/−^) Y strain, and *in vivo* using an experimental model of *T. cruzi* infection in BALB/c mice. Our *in-vitro* results showed a significant decrease in the host cell invasion rate by TcP21^−/−^ parasites as measured by Giemsa staining and cell count in bright light microscope. Quantitative polymerase chain reaction (qPCR) analysis showed that TcP21^−/−^ parasites multiplied intracellularly to a higher extent than the scrambled parasites at 72h post-infection. In addition, we observed a higher egress of TcP21^−/−^ trypomastigotes from C2C12 cells at 144h and 168h post-infection. Mice infected with Y strain TcP21^−/−^ trypomastigotes displayed higher systemic parasitemia, heart tissue parasite burden, and several histopathological alterations in heart tissues compared to control animals infected with scrambled parasites. Therewith, we propose that P21 is important in the host–pathogen interaction during invasion, cell multiplication, and egress, and may be part of the mechanism that controls parasitism and promotes chronic infection without patent systemic parasitemia.

## 1 Introduction

*Trypanosoma cruzi* (*T. cruzi*) is a flagellated protozoan endemic in Latin America and the etiological agent of Chagas disease. *T. cruzi* is morphologically characterized by having three distinct evolutionary stages: epimastigote, trypomastigote, and amastigote. Development from one stage to another is a complex process, involving ultrastructural, antigenic, and physiological changes (Brener, 2003; de Souza, 2007). During the process of cell invasion, infective forms of *T. cruzi* (metacyclic trypomastigote, bloodstream trypomastigote, and extracellular amastigote) use different molecules to interact with host cell components to overcome the obstacles imposed by the mammalian host.

The protein P21 binds to the host cell in a dose-dependent manner, is ubiquitously expressed and secreted, and is involved in host cell invasion by trypomastigotes and extracellular amastigotes (da Silva et al., 2009). The use of the recombinant form of P21 (rP21) revealed that the native protein may promote phagocytosis by binding to the CXCR4 receptor and has chemotactic activity for macrophages and neutrophils (Rodrigues et al., 2012). It has also been demonstrated that rP21-induced myeloperoxidase and IL-4 production and decreased blood vessel formation *in vitro* and *in vivo* (Teixeira et al., 2015). In addition, rP21 reduced the growth of epimastigotes, inhibited intracellular replication of amastigotes, and modulated the parasite cell cycle (Teixeira et al., 2019). Corroborating with these results, we observed that rP21 decreased the multiplication of *T. cruzi* (Y strain) in C2C12 myoblasts, a phenomenon associated with greater actin polymerization and higher expression of IL-4 (Martins et al., 2020).

We have generated parasites of Y strain that are knockout for P21 by CRISPR/Cas9. The ablation of P21 in these parasites inhibited epimastigotes multiplication and upregulated intracellular amastigotes replication in HeLa cells infected with metacyclic trypomastigotes. To assess potential additional roles of P21 in host cell invasion, multiplication, egress, and cardiac tissue parasite load, we used tissue culture-derived trypomastigotes (TCT) of Y strain that are knockout for P21 (TcP21^−/−^) to infect C2C12 cell line *in vitro* and BALB/c mice *in vivo*. The results showed that Y strain TcP21^−/−^ parasites invaded C2C12 cells to a lower extent, multiplied at higher levels at 72h post-infection, and egressed significantly more at 144h post-infection than scrambled parasites. *In vivo*, mice infected with Y strain TcP21^−/−^ parasites showed higher parasitemia, cardiac tissue parasite load, and cardiac tissue histopathological alterations than those infected with scrambled parasites.

## 2 Materials and methods

### 2.1 Parasite and cell cultures

Epimastigotes of Y (DTU II) strain were grown at 28°C in liver infusion tryptose (LIT) medium supplemented with 20% fetal bovine serum (FBS; Invitrogen). To differentiate epimastigotes into metacyclic forms, the epimastigotes were maintained in LIT for 14 days, and metacyclic trypomastigotes were purified as previously described (Teixeira and Yoshida, 1986).

Vero and C2C12 cells (obtained from Instituto Adolfo Lutz, São Paulo, SP, Brazil) were cultured in Dulbecco’s minimal essential medium (DMEM) (Sigma Chemical Co., St. Louis, MO, USA) supplemented with 10% FBS (Cultilab, Campinas, SP, Brazil), 10 μg/ml streptomycin, 100 U/ml penicillin, and 40 μg/ml gentamycin at 37°C in a 5% CO_2_ humid atmosphere.

Cultures of scrambled and TcP21^−/−^ epimastigotes of Y strain in the stationary phase containing metacyclic trypomastigotes (Teixeira et al., 2022) were used to infect Vero cells to obtain TCT forms for *in-vitro* and *in-vivo* experiments.

### 2.2 Animals and ethics

Six- to eight-week-old male BALB/c mice (15 animals) were maintained under standard conditions on a 12h light-dark cycle in a temperature-controlled setting (25°C), with food and water *ad-libitum*. Maintenance and animal care complied with the guidelines of the Ethics Committee for the Use of Animals (CEUA). Animal euthanasia was performed based on international welfare grounds according to the American Veterinary Medical Association Guidelines on Euthanasia. For euthanasia, mice were anesthetized intraperitoneally with a solution containing ketamine hydrochloride (100 mg/kg) and xylazine hydrochloride (10 mg/kg) followed by cervical dislocation. This study was approved by CEUA-UFU, with protocol number: 23117.077543/2022–27.

### 2.3 Biosafety approval

This study was approved by the CTNBio for the use of genetically modified organisms with the process number: 01245.004217/2023–83 and extract number: 8739/2023.

### 2.4 Host cell invasion and egress assays

C2C12 cell invasion assay was performed by adding 500 μl of cell suspension (5 × 10^4^ cells and 3 × 10^4^, respectively) into 24 well plates containing sterile glass coverslips (13 mm) and left seeding overnight. TCT suspensions of scrambled and Y strain TcP21^−/−^ parasites were added at a multiplicity of infection of 5 (five parasites per cell), and plates were incubated for 2h at 37°C in a CO_2_ (5%) humidified incubator. After incubation, cells were gently washed 3 times with phosphate-buffered saline, fixed with Bouin, and stained with Giemsa. The number of internalized parasites were counted in a total of 300 cells.

For the egress assay, which follows the host cell invasion described above and determines the number of parasites that exit the cells post-infection, plates were washed after infection and complete medium replaced, and then they were incubated at 37°C in a CO_2_ (5%) humidified incubator. After 72h and up to 10 days (240h) post-infection, the number of parasites in the supernatant was determined by counting trypomastigote and amastigote forms using a Neubauer chamber.

These experiments were performed in three technical replicates and three independent biological procedures.

### 2.5 *In-vivo* infection

BALB/c mice were randomized into three groups, each containing five mice. Group 1: animals not infected; Group 2: animals infected with scrambled parasites (control); and Group 3: animals infected with TcP21^−/−^ parasites. Both scrambled and TcP21^−/−^ were parasites of Y strain.

Animals were infected intraperitoneally with 10^5^ parasites. A systemic parasitemia was determined from day 3 post-infection, and then every other day up to day 15 post-infection, by collecting 5 µl of blood from the animal’s tail, and the parasites were counted under light microscopy. On day 15 post-infection, after performing parasitemia, animals were euthanized and their hearts were collected for histopathological analysis and quantification of parasite DNA by qPCR.

### 2.6 Parasite load determined by qPCR

The hearts collected after euthanasia were weighed (100 mg) and stored in liquid nitrogen. After maceration with the aid of a porcelain crucible, a lysis buffer containing 500 μl of nuclei lysis buffer, 16 μl of Sodium dodecyl-sulfate (SDS) at 10%, and 8 μl of Proteinase K solution was added following an incubation at 50°C overnight. Next, 150 μl of NaCl buffer was added to the lysed hearts, which were then vortexed for 15 s and placed on ice for 10 min. The supernatant was collected and transferred to an Eppendorf tube. After addition of 800 μl of absolute ethanol solution, the tube was mixed well by inversion and centrifuged at 12000 rpm for 15 min. After discarding the supernatant, 1 mL of ethanol (75%) was added to the sample pellet, mixed and centrifuged again at 12000 rpm for 5 min. The supernatant was discarded and the sample pellet was allowed to dry for 10 min. The pellet was resuspended with 15–200 μl of RNAse and DNAse free water.

The DNA was quantified by nanodrop and a quantitative PCR was performed on the ABI Prism 7500 Fast System (Applied Biosystems, Foster City, CA) using a final sample volume of 10 μl [4 μl of DNA, 5 μl Power SYBR Green PCR Master Mix (Applied Biosystems, Foster City, CA, EUA) and 1 μl of primers Diaz7 e Diaz8 (Diaz et al., 1992)].

The standard curve was obtained using serial dilutions of 100ng of DNA extracted from epimastigotes with a limit of 0.0001 fg as proposed by Diaz et al. (1992) (Diaz et al., 1992) and modified by De Oliveira et al. (2020) (de Oliveira et al., 2020). Positive, negative, and reagent internal controls were used in all qPCR reactions.

A similar procedure was applied to C2C12 cells infected with TCTs of Y strain in order to obtain the DNA from these cells during the kinetics of multiplication. In this case, a total of 1 × 10^5^ infected cells were harvested and lysed. The experiment was performed three times in triplicate.

### 2.7 Inflammatory score

Heart samples were fixed in 10% buffered formalin solution, dehydrated in ethanol solution, diaphanized in xylene, and embedded in paraffin. Blocks containing hearts were sectioned at 5-μm thick sections, and then placed onto glass slides and stained. To evaluate the number of amastigote nests, inflammatory infiltrate, and damage tissue score, slides of cardiac tissue were stained with hematoxylin and eosin (HE). The amastigote nests in each slide were qualitatively measured under light microscopy. The inflammatory infiltrate and damage tissue were scored by intensity: (−) absent, (+) mild, (++) moderate, and (+ + +) intense as described by Da Silva et al. (2018) (Da Silva et al., 2018).

### 2.8 Statistical analysis

All data were presented as the mean ± standard error (mean ± SEM) of at least three independent experiments performed in triplicate. The normal distribution of the data was checked using a Shapiro–Wilk test. Then, the significant differences were determined by *t*-test, and a multiple comparison by Mann–Whitney test. For some data, the significant differences were determined using a two-way analysis of variance (ANOVA), and the multiple comparison by Bonferroni’s test for parametric data and Sidak’s test for non-parametric data, value of *p* ≤ 0.05 were considered significant. All the statistical analyses were performed using GraphPad Prism software version 8.0.1.

## 3 Results

### 3.1 Knockout of P21 in TCT from *T. cruzi* of Y strain affects the cell invasion and multiplication

**Figure 1:**
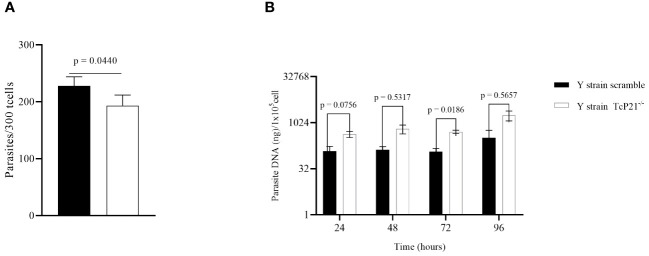
Cell invasion and intracellular multiplication of TcP21^−/−^ and scrambled *T. cruzi* of Y strain in C2C12 cell lines. **(A)** C2C12 cell invasion—number of intracellular parasites in 300 cells. TcP21^−/−^ parasites showed lower invasion rate compared to the scrambled group; **(B)** qPCR analysis showed that, at 72h post-infection, TcP21^−/−^ parasites showed a higher multiplication rate compared to the scrambled group. All data were analyzed using GraphPad Prism software version 8.0.1. The graph shows the mean ± SEM of three experiments performed in triplicate. The comparison of invasion data between the knockout (TcP21^−/−^) and control (scrambled) parasites was performed using *t*-test and Mann–Whitney test for multiple comparison. Statistical differences of intracellular multiplication were determined by two-way ANOVA and Bonferroni’s test for multiple comparisons. In all analyses, the value of *p* < 0.05 was considered to be statistically significant.

TcP21^−/−^ parasites showed a decrease in host cell invasion compared to control (scrambled) parasites in C2C12 cells (*p* = 0.0440) (**Figure 1A**). However, qPCR analysis showed that TcP21^−/−^ multiplied at a higher level than the scrambled parasites at 72h post-infection (*p* = 0.0186) (**Figure 1B**).

### 3.2 The knockout of P21 affected the egress of Y-strain parasites

**Figure 2:**
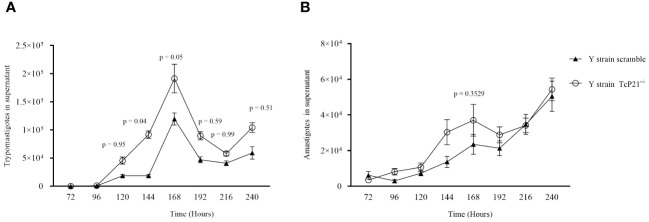
Egress of trypomastigotes and amastigotes from infected C2C12 cells along with the kinetics of parasite multiplication. **(A, B)** Egress of TcP21^−/−^ and scrambled trypomastigotes and amastigotes of Y strain from C2C12 cells. All data were analyzed using GraphPad Prism software version 8.0.1. The graph shows the mean ± SEM of three experiments performed in triplicate. Statistical differences between the egress of knockout (TcP21^−/−^) and control (scrambled) trypomastigotes and amastigotes were analyzed using two-way ANOVA and Sidak’s test for multiple comparisons. In all analyzes the value of *p* < 0.05 was considered to be statistically significant.

Regarding egress of trypomastigotes and amastigotes of Y-strain, we observed a higher number of TcP21^−/−^ trypomastigotes in the supernatant of infected cells at 144 (*p* = 0.0400) and 168 (*p* = 0.0500) hours post-infection compared to scrambled trypomastigotes (**Figure 2A**). The release of amastigotes to the supernatant of C2C12 cells was similar between both groups (**Figure 2B**).

### 3.3 The knockout of P21 affected the parasitism in mice infected with Y strain

**Figure 3:**
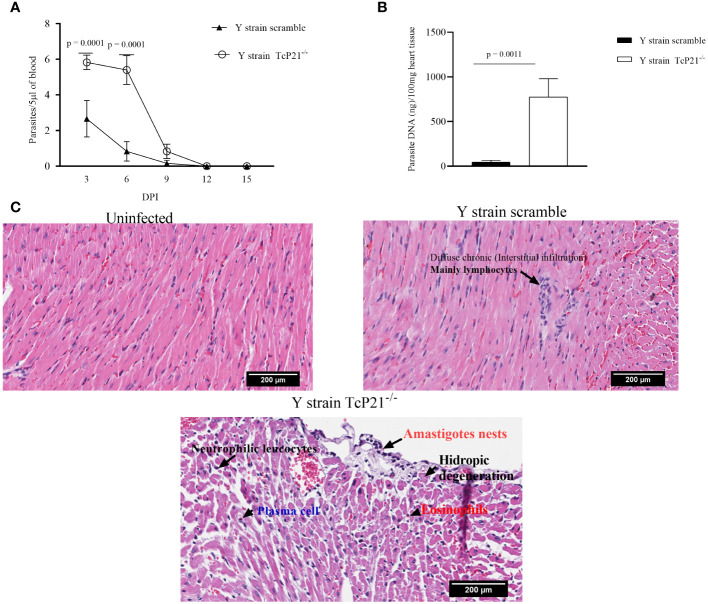
Systemic parasitism and Cardiac parasite load. **(A)** Systemic parasitism detected every other day along 15 days of BALB/c mice infection; **(B)** cardiac parasite load of BALB/c mice infected with parasites of Y strain; **(C)** representative images of the cardiac tissue inflammatory score of BALB/c mice infected with *T. cruzi* of Y strain. The histopathological alterations are indicated by black arrows. *n* = 5 animals per group. All data were analyzed using GraphPad Prism software version 8.0.1. The graph shows the mean ± SEM of three experiments performed in triplicate. The comparison of heart tissue parasite load data between the knockout (TcP21^−/−^) and control (scrambled) parasites was performed using unpaired *t*-test for multiple comparisons. In all analyses, the value of *p* < 0.05 was considered to be statistically significant. Bars: 200µm.

Animals infected with Y strain TcP21^−/−^ showed a significantly higher systemic parasitemia compared to animals infected with scrambled parasites at days 3 and 6 post-infection (*p* = 0.0001) (**Figure 3A**). When heart samples were analyzed for parasite load by qPCR, we observed a higher parasite burden in animals infected with Y strain TcP21^−/−^ parasites than mice infected with scrambled ones (*p* = 0.0011) (**Figure 3B**).

### 3.4 The knockout of P21 affected the inflammatory score in heart tissue from mice infected with TCT of Y strain

A qualitative analysis of heart tissue from animals infected with Y strain TcP21^−/−^ parasites showed increased presence of neutrophilic leucocytes, eosinophils, macrophages, tissue damage, apoptotic bodies, and amastigote nests compared to tissues from mice infected with scrambled parasites (**Table 1**). Representative images are shown in **Figure 3C**.

**Table 1:** Qualitative histological analyses of heart tissues from BALB/c mice at 15 days post-infection with TCT of *T. cruzi (*Y strain).

Histological criteria	Non infected (*n* = 5)	Scrambled (*n* = 5)	TcP21^−/−^ (*n* = 5)
Inflammatory response	None	Low/mild	Moderate
Predominantly	–	Diffuse chronic (interstitial infiltration)	Diffuse chronic (interstitial infiltration)
Neutrophilic leucocytes	–	+	++
Eosinophils	–	–	+
Macrophages	–	+	++
Lymphocytes	–	++	+++
Plasma cells	–	+	+
Giant foreign body cells	–	–	–
Tissue damage	–	+	++
Hydropic degeneration	–	++	+++
Necrotic tissue	–	–	–
Apoptotic bodies	–	–	+
Edema	–	+	+++
Fibroblast	–	+	+
Fibrosis	–	+	+
Adipocyte	–	–	–
Epicardium calcification	–	–	–
Amastigotes nests	–	+	++

(−) absent, (+) mild, (++) moderate, and (+ + +) intense.

## 4 Discussion

Our previous results using the recombinant form of P21 (rP21) have suggested that this *T. cruzi* ubiquitous and specific protein plays a role in the invasion and multiplication processes of the infective forms of the parasite. Although P21 is not conserved among eukaryotic species, we observed that its recombinant form induces phagocytosis of *Leishmania amazonensis* and *Toxoplasma gondii* (Rodrigues et al., 2012). In addition, we observed that rP21 induces *T. gondii* invasion and decreases its multiplication in BeWo cell line (de Souza et al., 2023).

In this scenario, P21 may be involved in the modulation of host cell invasion by the parasite, in its multiplication and egress from host cells. Therefore, we proposed that P21 maintains parasites intracellularly at low multiplication rate and away from host immune attack, leading the disease to the chronic phase without systemic parasitemia. In order to confirm this hypothesis, we first verified the impact of knocking out P21 from metacyclic trypomastigotes on the host cell invasion and multiplication using HeLa cell line *in vitro*. Corroborating with our hypothesis, results showed that the P21 knockout impaired parasite host cell invasion and induced parasite multiplication at 72h post-infection (Teixeira et al., 2022).

Here, we addressed the extended impact of knocking out P21 from the virulent strain (Y strain–DTU II) on cell invasion, multiplication, egress, systemic parasitemia, and cardiac tissue parasite load. The infective form used was TCT and the host cell was C2C12 cell line. Our results showed that Y strain TcP21^−/−^ parasites invaded C2C12 cells at a lower rate than control (scrambled) parasites. These results confirmed the findings of our recently published study performed with knockout metacyclic trypomastigotes from the same strain (Teixeira et al., 2022).

qPCR procedure showed higher multiplication rate at 72h post-infection for TcP21^−/−^parasites in comparison to scrambled ones. This is consistent with our previously raised hypothesis and with our recent study using metacyclic trypomastigotes of Y strain (Teixeira et al., 2022). The number of trypomastigotes in the supernatant was significantly higher in C2C12 cells infected by the knockout parasites compared to scrambled parasites at 144h and 168h post-infection. The higher egress of knockout parasites compared to scrambled ones may reflect the higher ability of these parasites to differentiate back into trypomastigote forms.

In order to verify the impact of P21 knockout in a complex system, we infected BALB/c mice with these parasites. Animals infected with Y strain TcP21^−/−^ parasites showed higher systemic parasitemia and a higher parasite load in heart tissues compared to animals infected with scrambled parasites. This is the first time that we confirmed the ability of P21 in controlling the infectivity of the parasite *in vivo*, reinforcing our *in-vitro* data. The observed greater infection rate *in vivo* led by the absence of P21 highlights the role of P21 on host parasitism, which is likely a consequence of the combined effects of P21 observed *in vitro* such as the ability of the parasite to invade, multiply, differentiate, and egress from the host cells. Therefore, it is plausible to suggest that P21 may play an important role in the control of parasitism of Y-strain parasites.

The histopathological analysis of hearts obtained from mice infected with Y strain TcP21^−/−^ parasites showed several pathological alterations, including presence of neutrophil and macrophage infiltrates, apoptotic bodies, and a high number of amastigotes nests. These results further support P21 as an important player in controlling infection by virulent strains in order to establish a chronic infection without much damage to the host.

Recently, authors have demonstrated that *T. cruzi* can enter a state of spontaneous dormancy. The dormant amastigotes are highly resistant to therapy both *in vivo* and *in vitro* (Sánchez-Valdéz et al., 2018). In addition, authors have shown the existence of an adaptive difference between *T. cruzi* strains to generate dormant cells, and that homologous recombination may be important for dormancy (Resende et al., 2020). Conversely, another research group suggested that *T. cruzi* persistence continues to involve regular cycles of replication, host cell lysis, and re-infection. They could find no evidence for wide-spread dormancy in parasites that persist in tissue reservoir (Ward et al., 2020). Although the dormancy in *T. cruzi* is still a matter of debates, we believe that P21 takes place in a machinery involved in the control of parasite multiplication, leading the disease to the chronic phase.

## 5 Conclusion

We conclude that some finely regulated mechanisms control parasite multiplication, differentiation, and egress during infection. As our results showed that P21 plays a role in cell invasion, intracellular multiplication, and egress *in vitro* and in the parasitism in *in-vivo* experiments, we propose that P21 may be a protagonist in the machinery that would be involved in the perpetuation of the disease in the infected host, since it seems to orchestrate the intracellular retention of the parasite from the virulent Y strain.

## Data availability statement

The raw data supporting the conclusions of this article will be made available by the authors, without undue reservation.

## Ethics statement

The animal study was approved by Comissão de Ética na Utilização de Animais/Universidade Federal de Uberlândia. The study was conducted in accordance with the local legislation and institutional requirements.

## Author contributions

CS: Conceptualization, Data curation, Formal analysis, Funding acquisition, Project administration, Supervision, Writing – original draft, Writing – review & editing. AS: Investigation, Writing – original draft. NU: Investigation, Writing – original draft. TV: Investigation, Writing – original draft. BB: Investigation, Writing – original draft. TT: Investigation, Writing – original draft. VA: Investigation, Writing – original draft. JT: Investigation, Writing – original draft. CP: Investigation, Writing – original draft. GS: Investigation, Writing – original draft. ST: Investigation, Writing – original draft. JS: Investigation, Writing – original draft. MS: Methodology, Resources, Writing – original draft. TM: Methodology, Resources, Writing – original draft. RR: Methodology, Resources, Writing – original draft. RM: Funding acquisition, Project administration, Writing – original draft, Writing – review & editing. JDS: Funding acquisition, Project administration, Writing – original draft, Writing – review & editing.

## References

[B1] Brener Z. (2003). Biology of trypanosoma cruzi. Ann Rev Microbiol. 27, 347–382. doi: 10.1146/ANNUREV.MI.27.100173.002023 4201691

[B2] Da Silva M. V. De Almeida V. L. De Oliveira W. D. Matos Cascudo N. C. De Oliveira P. G. Da Silva C. A.. (2018). Upregulation of Cardiac IL-10 and Downregulation of IFN- γ in Balb/c IL-4-/-in Acute Chagasic Myocarditis due to Colombian Strain of *Trypanosoma cruzi* . Mediators Inflammation 2018. doi: 10.1155/2018/3421897 PMC6304210 30622430

[B3] da Silva C. V. Kawashita S. Y. Probst C. M. Dallagiovanna B. Cruz M. C. da Silva E. A.. (2009). Characterization of a 21 kDa protein from *Trypanosoma cruzi* associated with mammalian cell invasion. Microbes Infect. 11, 563–570. doi: 10.1016/j.micinf.2009.03.007 19344784

[B4] de Oliveira M. T. Sulleiro E. Gimenez A. S. de Lana M. Zingales B. da Silva J. S.. (2020). Quantification of parasite burden of *Trypanosoma cruzi* and identification of Discrete Typing Units (DTUs) in blood samples of Latin American immigrants residing in Barcelona, Spain. PLoS Negl. Trop. Dis. 14, e0008311. doi: 10.1371/JOURNAL.PNTD.0008311 32497037 PMC7271996

[B5] de Souza W. (2007). Chagas’ disease: facts and reality. Microbes Infect. 9, 544–545. doi: 10.1016/j.micinf.2006.12.014 17336119

[B6] de Souza G. Teixeira S. C. Fajardo Martínez A. F. Silva R. J. Luz L. C. de L. JúniorJ. P.. (2023). *Trypanosoma cruzi* P21 recombinant protein modulates *Toxoplasma gondii* infection in different experimental models of the human maternal-fetal interface. Front. Immunol. 14. doi: 10.3389/fimmu.2023.1243480 PMC10617204 37915581

[B7] Diaz C. Nussenzweig V. Gonzalez A. (1992). An improved polymerase chain reaction assay to detect *Trypanosoma cruzi* in blood. Am. J. Trop. Med. Hyg 46, 616–623. doi: 10.4269/ajtmh.1992.46.616 1599057

[B8] Martins F. A. dos Santos M. A. Santos J de G. da Silva A. A. Borges B. C. da Costa M. S.. (2020). The recombinant form of *trypanosoma cruzi* P21 controls infection by modulating host immune response. Front. Immunol. 11. doi: 10.3389/fimmu.2020.01010 PMC7325895 32655546

[B9] Resende B. C. Oliveira A. C. S. Guañabens A. C. P. Repolês B. M. Santana V. Hiraiwa P. M.. (2020). The influence of recombinational processes to induce dormancy in trypanosoma cruzi. Front. Cell Infect. Microbiol. 10. doi: 10.3389/fcimb.2020.00005 PMC7025536 32117793

[B10] Rodrigues A. A. Clemente T. M. dos Santos M. A. MaChado F. C. Gomes R. G. B. Moreira H. H. T.. (2012). A recombinant protein based on *trypanosoma cruzi* P21 enhances phagocytosis. PLoS One 7, e51384. doi: 10.1371/journal.pone.0051384 23251513 PMC3519637

[B11] Sánchez-Valdéz F. J. Padilla A. Wang W. Orr D. Tarleton R. L. (2018). Spontaneous dormancy protects Trypanosoma cruzi during extended drug exposure. Elife 7. doi: 10.7554/eLife.34039 PMC5906098 29578409

[B12] Teixeira T. L. Castilhos P. Rodrigues C. C. da Silva A. A. Brígido R. T. Teixeira S. C.. (2019). Experimental evidences that P21 protein controls Trypanosoma cruzi replication and modulates the pathogenesis of infection. Microb. Pathog. 135. doi: 10.1016/j.micpath.2019.103618 31310832

[B13] Teixeira T. L. Chiurillo M. A. Lander N. Rodrigues C. C. Onofre T. S. Ferreira E. R.. (2022). Ablation of the P21 gene of *trypanosoma cruzi* provides evidence of P21 as a mediator in the control of epimastigote and intracellular amastigote replication. Front. Cell Infect. Microbiol. 12. doi: 10.3389/fcimb.2022.799668 PMC8895596 35252026

[B14] Teixeira T. L. MaChado F. C. Alves Da Silva A. Teixeira S. C. Borges B. C. Dos Santos M. A.. (2015). *Trypanosoma cruzi* P21: a potential novel target for chagasic cardiomyopathy therapy. Sci. Rep. 5, 1 2015. doi: 10.1038/srep16877 PMC4648062 26574156

[B15] Teixeira M. M. G. Yoshida N. (1986). Stage-specific surface antigens of metacyclic trypomastigotes of *Trypanosoma cruzi* identified by monoclonal antibodies. Mol. Biochem. Parasitol. 18, 271–282. doi: 10.1016/0166-6851(86)90085-X 3515178

[B16] Ward A. I. Olmo F. Atherton R. L. Taylor M. C. Kelly J. M. (2020). *Trypanosoma cruzi* amastigotes that persist in the colon during chronic stage murine infections have a reduced replication rate: *Trypanosoma cruzi* proliferation. Open Biol. 10. doi: 10.1098/rsob.200261 PMC7776577 33321060

